# Radiation and immune checkpoint inhibitor-mediated pneumonitis risk stratification in patients with locally advanced non-small cell lung cancer: role of functional lung radiomics?

**DOI:** 10.1007/s12672-022-00548-4

**Published:** 2022-09-01

**Authors:** Hannah M. T. Thomas, Daniel S. Hippe, Parisa Forouzannezhad, Balu Krishna Sasidharan, Paul E. Kinahan, Robert S. Miyaoka, Hubert J. Vesselle, Ramesh Rengan, Jing Zeng, Stephen R. Bowen

**Affiliations:** 1 Department of Radiation Oncology, University of Washington School of Medicine, 1959 NE Pacific St, Box 356043, Seattle, WA 98195 USA; 2 Department of Radiation Oncology, Christian Medical College Vellore, Vellore, Tamil Nadu India; 3 Clinical Research Division, Fred Hutchinson Cancer Center, Seattle, WA USA; 4 Department of Radiology, University of Washington School of Medicine, Seattle, WA USA

**Keywords:** Functional lung imaging, Radiomics, SPECT, Pneumonitis, Radiation therapy, Immunotherapy, Machine learning

## Abstract

**Background:**

Patients undergoing chemoradiation and immune checkpoint inhibitor (ICI) therapy for locally advanced non-small cell lung cancer (NSCLC) experience pulmonary toxicity at higher rates than historical reports. Identifying biomarkers beyond conventional clinical factors and radiation dosimetry is especially relevant in the modern cancer immunotherapy era. We investigated the role of novel functional lung radiomics, relative to functional lung dosimetry and clinical characteristics, for pneumonitis risk stratification in locally advanced NSCLC.

**Methods:**

Patients with locally advanced NSCLC were prospectively enrolled on the FLARE-RT trial (NCT02773238). All received concurrent chemoradiation using functional lung avoidance planning, while approximately half received consolidation durvalumab ICI. Within tumour-subtracted lung regions, 110 radiomics features (size, shape, intensity, texture) were extracted on pre-treatment [^99m^Tc]MAA SPECT/CT perfusion images using fixed-bin-width discretization. The performance of functional lung radiomics for pneumonitis (CTCAE v4 grade 2 or higher) risk stratification was benchmarked against previously reported lung dosimetric parameters and clinical risk factors. Multivariate least absolute shrinkage and selection operator Cox models of time-varying pneumonitis risk were constructed, and prediction performance was evaluated using optimism-adjusted concordance index (c-index) with 95% confidence interval reporting throughout.

**Results:**

Thirty-nine patients were included in the study and pneumonitis occurred in 16/39 (41%) patients. Among clinical characteristics and anatomic/functional lung dosimetry variables, only the presence of baseline chronic obstructive pulmonary disease (COPD) was significantly associated with the development of pneumonitis (HR 4.59 [1.69–12.49]) and served as the primary prediction benchmark model (c-index 0.69 [0.59–0.80]). Discrimination of time-varying pneumonitis risk was numerically higher when combining COPD with perfused lung radiomics size (c-index 0.77 [0.65–0.88]) or shape feature classes (c-index 0.79 [0.66–0.91]) but did not reach statistical significance compared to benchmark models (p > 0.26). COPD was associated with perfused lung radiomics size features, including patients with larger lung volumes (AUC 0.75 [0.59–0.91]). Perfused lung radiomic texture features were correlated with lung volume (adj R^2^ = 0.84–1.00), representing surrogates rather than independent predictors of pneumonitis risk.

**Conclusions:**

In patients undergoing chemoradiation with functional lung avoidance therapy and optional consolidative immune checkpoint inhibitor therapy for locally advanced NSCLC, the strongest predictor of pneumonitis was the presence of baseline chronic obstructive pulmonary disease. Results from this novel functional lung radiomics exploratory study can inform future validation studies to refine pneumonitis risk models following combinations of radiation and immunotherapy. Our results support functional lung radiomics as surrogates of COPD for non-invasive monitoring during and after treatment. Further study of clinical, dosimetric, and radiomic feature combinations for radiation and immune-mediated pneumonitis risk stratification in a larger patient population is warranted.

**Supplementary Information:**

The online version contains supplementary material available at 10.1007/s12672-022-00548-4.

## Introduction

Pneumonitis is a well reported adverse effect of lung-directed radiation and immune checkpoint inhibitor (ICI) therapy independently [1–6] and it is also recognized that ICI may have an additive effect to pneumonitis caused by radiation therapy [7, 8]. Clinically symptomatic radiation pneumonitis manifests in 30–40% of patient within weeks to months following concurrent chemoradiation regimens, with most cases being reported within the first 6–8 months [9]. Based on the outcome of the PACIFIC trial [10], the standard-of-care for treating unresectable locally advanced non-small cell lung cancer (NSCLC) has evolved to include consolidation anti-PD-L1 (monoclonal antibody targeted against programmed cell death-1 ligand 1 (PD-L1) ICI therapy, which conferred a clinically meaningful survival advantage when administered after chemoradiation [11]. Although pneumonitis incidence varies widely based on a multitude of risk factors [4, 12, 13], this regimen has been shown to increase pneumonitis incidence and frequency treatment interruptions in some patients, potentially blunting clinical benefits [6, 10, 14–17]. The interruptions in patients on ICI therapy often includes withholding ICI, starting steroids, and if needed, adding immunosuppressants. A prolonged period of corticosteroid treatment and slow tapering is also required due to the extended half-life of the antibodies [18].

Predictive risk models of radiation pneumonitis are primarily based on clinical parameters and radiation dosimetry. Reported risk factors include advanced age, gender, performance status, history of smoking, tumour location, underlying pulmonary comorbidities, radiation treatment regimen, and concurrent chemotherapy [19–22]. Common radiation dosimetric predictors include mean lung dose (MLD), dose-volumes (V20, V30, V40), total radiation lung dose, and daily radiation dose [19, 23–27]. Other risk factors reported include race, dose to the heart and trachea or bronchus [21, 28, 29]. These risk factors can be combined in normal tissue complication probability models with weighted non-parametric decision trees [28, 29].

Beyond conventional radiation dosimetry, functional lung dosimetry derived from positron emission tomography (PET), four-dimensional respiratory-correlated CT (4DCT), single photon emission computed tomography (SPECT) or magnetic resonance (MR) imaging can be used to limit dose to well-perfused or well-ventilated lung under functional tissue avoidance planning paradigms [30–37]. Functional lung dosimetric predictors of pneumonitis include functional MLD, functional V5, V20, V30, V40, and total lung perfusion receiving dose above 20 Gy [24, 38–41]. Other quantitative imaging biomarkers, based primarily on CT radiomic texture features, have also improved pneumonitis risk stratification relative to traditional dosimetry [42–46].

Unlike validated radiation pneumonitis modelling, identifying risk factors for combined radiation and ICI-mediated pneumonitis is plagued by a paucity of data and limited diagnostic accuracy based on specific clinical or radiographic markers [47]. Inoue et al. reported that typical clinical and dose-volume parameters failed to show correlation to pneumonitis following radiation therapy (RT) and anti-PD-L1 ICI [48]. Modern chemoradiation and ICI-therapy regimens require discovery of novel biomarkers that can capture differential presentations of radiation and immune-mediated pneumonitis [6] at overlapping time intervals.

We investigated the utility of functional lung radiomics for pneumonitis risk stratification in the setting of chemoradiation and ICI-therapy for patients with LA-NSCLC enrolled on a prospective clinical trial of functional lung avoidance and response-adaptive escalation (FLARE) RT. Our aim is to explore the role of functional lung radiomics, relative to functional lung dosimetry and clinical characteristics, for pneumonitis risk stratification in LA-NSCLC.

## Methods and material

### Patient population and treatment characteristics

Patients with histologically confirmed LA-NSCLC enrolled onto the prospective FLARE-RT clinical trial were included in this study. Patients without a baseline and 3-month post-treatment SPECT and PET imaging, and follow-up of at least 3 months were excluded from the study. All patients received concurrent chemotherapy. Functional lung avoidance radiation treatment was planned to limit dose to well-perfused lung regions defined on ^99m^Tc-labled macro-aggregated albumin (MAA) SPECT. Adaptive dose escalation was planned to residual FDG-PET-avid regions in select patients based on mid-treatment response assessment [49, 50]. Mid-PET responders and non-responders were prescribed doses of 60 Gy and 74 Gy, respectively, to the planning target volumes. Approximately half of the patients (20/39) received pencil beam scanned proton therapy, while the remaining patients received photon IMRT/VMAT. Following the transition to a new standard-of-care pathway, 21/39 patients received consolidative durvalumab anti-PD-L1 ICI-therapy after completion of chemoradiation.

Patients were seen at least once every 6 weeks during the first 3 months after radiation, and at least once every 3 months for the first two years. Patients receiving durvalumab after radiation were seen every two weeks during their infusions for assessment. Patient were seen for additional clinical visits as needed to manage clinical symptoms. All patients presenting with worsening respiratory symptoms, including changes in dyspnoea relative to baseline, underwent CT chest imaging. Adequate medical evaluation was done to rule out other differential causes for the lung findings. Pneumonitis was confirmed by the multi-disciplinary team based on CT imaging and by diagnosis of exclusion. Based on the imaging results, patients were sometimes treated with an initial course of antibiotics for potential bacterial pneumonia. If antibiotics did not lead to resolution of symptoms, patients were then placed on steroids, if the symptoms were significant enough to require treatment. Clinical grade pneumonitis incidence was dated at the start of symptoms. Patients were dichotomized for pulmonary toxicity based on the presence or absence of grade ≥ 2 pneumonitis (GR2 + pneumonitis) defined by CTCAE v4. Due to the brief time interval between completion of chemoradiation and initiation of consolidation durvalumab in most patients, we did not distinguish between radiation pneumonitis and immune-mediated pneumonitis events. The study was reviewed and approved by the institutional review board.

### Image acquisition and processing

Patients underwent pre-treatment [^99^mTc] MAA perfusion SPECT/CT on a Precedence (Philips Healthcare, Cleveland, OH) 16-slice CT scanner with a dual-head gamma camera to extract candidate radiomic predictors of pneumonitis. All imaging data was acquired with patients reproducibly immobilized in radiation treatment position. Patients received a fixed intravenous injected activity (185 MBq nominal), followed by a time-averaged SPECT (64 views, 20 s per view, 180-degree arc) acquired under quiescent free-breathing conditions. SPECT images were corrected for scatter, collimator-detector response, and attenuation using helical CT images. Images were reconstructed using the Astonish™ (Philips Healthcare, Cleveland, OH) ordered subset expectation–maximization (OSEM) iterative algorithm on 4.64 mm isotropic grids with spatial resolution recovery and 10 mm cut-off Hanning filter. The CT elements of SPECT/CT pre-and post-treatment images were rigidly co-registered to planning 4DCT average intensity projection images in MIM 6.8™ (MIM Software Inc., Cleveland, OH) using mutual information. The spatial transformations estimated from CT-to-CT registration were subsequently applied to the respective SPECT images.

### Lung ROI definition and dosimetry

Internal gross tumour volumes (IGTV) were delineated as the union of contours from respiratory-correlated 4DCT phases by board-certified radiation oncologists. The normal lung was defined as the Boolean subtraction of internal gross tumour volume from the lung (Lung-IGTV) on the planning 4DCT average intensity projection. Using the linear-quadratic model, the radiation dose to the normal lung was converted to 2 Gy fraction biologically equivalent voxel dose distributions (EQD2Lung) for a clinical endpoint of pneumonitis (α/β = 3) to account for variability in fractional dose across patients. All proton therapy doses were calculated with a commercial Monte-Carlo treatment planning algorithm appropriate for lung tissue [51, 52] and a constant radiobiological effectiveness (RBE) of 1.1. From the Lung-IGTV ROI, we extracted five previously reported EQD2Lung dosimetry parameters: (i) mean lung dose (MLD), (ii) volume of lung receiving ≥ 20 Gy (V20), (iii) perfused mean lung dose (pMLD), (iv) perfused lung volume receiving ≥ 20 Gy (pV20), and (v) fraction of integral lung function receiving ≥ 20 Gy (pF20). These metrics were identified as predictors for pneumonitis in patients undergoing conventional radiation therapy for NSCLC [35].

### Perfusion radiomics feature extraction

**Fig. 1:**
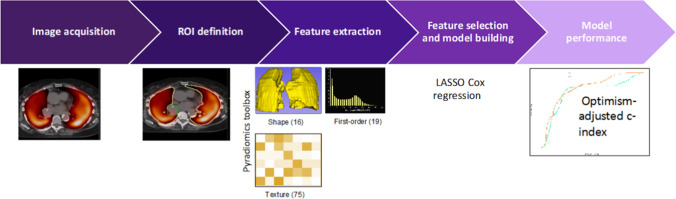
Functional lung radiomics and machine learning pipeline for pneumonitis risk stratification

Pre-treatment MAA SPECT perfusion images, co-registered 3-month post-treatment MAA SPECT perfusion images, and co-registered normal lung RT structures were loaded in 3D Slicer [53]. Within the Lung-IGTV contours, 110 features were extracted using the Radiomics module made available by the PyRadiomics community [54] (Fig. 1). The features were extracted at the native voxel size (4.64 mm isotropic) without any resampling or filtering, yielding a median of 34,644 voxels (range 20,043–78,484 voxels) per Lung-IGTV contour. We used fixed bin width (FBW = 25 CNTS) SPECT intensity discretization techniques which produced discrete intensity bins with sufficient voxel sampling for neighborhood texture calculation [54]. In all, 19 first-order, 16 shape and 75 texture features were computed from the grey-level co-occurrence (GLCM), grey-level dependence (GLDM), grey-level run length (GLRLM), grey-level size zone (GLSZM) and neighbourhood grey-tone difference (NGTDM) matrices. The GLCM was calculated using the PyRadiomics default symmetrical setting. Wavelet filtered features were not computed to limit dimensionality in this discovery phase study with modest patient sample size.

### Statistical analysis

Clinical and dosimetric parameters were compared between groups using Fisher’s exact test for categorical variables and the Mann–Whitney U‐test for continuous variables. Associations of clinical characteristics and dosimetric parameters with GR2 + pneumonitis were estimated using Cox regression models, with time to GR2 + pneumonitis censored by distant metastasis and death. Clinical characteristics in Table 1 with small sample sizes or a large number of missing values were not included in this analysis. ICI therapy was treated as a binary time-varying covariate in the models that was initialized to 0 (has not started therapy) and became 1 when therapy was initiated. The correlation of candidate pre-treatment functional lung radiomic biomarkers with clinical variables were performed using Spearman rank correlation and Mann–Whitney tests.

**Table 1:** Patient demographics and clinical characteristics stratified by pneumonitis status

Characteristics	All (N = 39)	GR2 + Pneumonitis	
		No (N = 23)	Yes (N = 16)
Age, years	63 (48–78)	62 (48–76)	67 (52–78)
Gender			
Male	18 (46)	10 (43)	8 (50)
Female	21 (54)	13 (57)	8 (50)
Clinical stage			
IIB	1 (3)	0 (0)	1 (6)
IIIA	19 (49)	9 (39)	10 (62)
IIIB	13 (33)	9 (39)	4 (25)
Recurrence	6 (15)	5 (22)	1 (6)
Treatment modality			
IMRT/VMAT	19 (49)	11 (48)	8 (50)
PBT	20 (51)	12 (52)	8 (50)
NSCLC histology			
Squamous cell carcinoma	14 (36)	6 (26)	8 (50)
Adenocarcinoma	23 (59)	16 (70)	7 (44)
NOS	2 (5)	1 (4)	1 (6)
COPD			
Yes	13 (33)	4 (17)	9 (56)
No	26 (67)	19 (83)	7 (44)
Chemotherapy			
Carboplatin-paclitaxel	23 (59)	12 (52)	11 (69)
Others	16 (41)	11 (48)	5 (31)
Immunotherapy			
Yes	21 (54)	11 (48)	10 (62)
No	18 (46)	12 (52)	6 (38)
Smoking status			
Non-smoker	5 (13)	3 (13)	2 (12)
Former	30 (77)	16 (70)	14 (88)
Current	4 (10)	4 (17)	0 (0)
PD-L1 tumor proportion score			
> 50%	5 (13)	3 (13)	2 (12)
1–49%	6 (15)	3 (13)	3 (19)
< 1%	4 (10)	3 (13)	1 (6)
Unknown	24 (62)	14 (61)	10 (62)
Mid-treatment PET response			
Responder	25 (64)	14 (61)	11 (69)
ICI-therapy	15 (60)	9 (64)	6 (55)
No ICI-therapy	10 (40)	5 (36)	5 (45)
Non-responder	14 (36)	9 (39)	5 (31)
ICI-therapy	6 (43)	2 (22)	4 (80)
No ICI-therapy	8 (57)	7 (78)	1 (20)

Values are median (range) or no (%)

*IMRT* intensity-modulated radiation therapy, *VMAT* volumetric modulated arc therapy, *PBT* proton-beam therapy, *NSCLC* non-small cell lung cancer, *NOS* not otherwise specified, *COPD* chronic obstructive pulmonary disease, *PD-L1* programmed death-ligand 1, *ICI* immune-checkpoint inhibitor

The least absolute shrinkage and selection operator (LASSO) was used for multivariable modeling. The LASSO was selected as the machine learning technique as it simultaneously performs both feature selection and parameter regularization to limit overfitting [55]. LASSO-Cox models were used for predicting GR2 + pneumonitis risk and LASSO-logistic models were used for evaluating associations with COPD at baseline (Table A2 and A3). Six primary models were generated for each outcome based on (1) clinical parameters only, (2) lung dosimetry parameters only (3) combination of both clinical and lung dosimetry parameters (4) radiomics feature class only, and (5) combination of radiomics feature classes and significant clinical feature (6) volume and all parameters from (5). The prediction performance of the models was evaluated using the concordance index (c-index) [56]. The c-index was optimism-adjusted using the bootstrap, an internal validation technique to account for training and testing models using the same data set [57].

All statistical calculations were performed using the statistical computing language R (version 4.0.3; R Foundation for Statistical Computing, Vienna, Austria). Throughout, two-sided tests were used, and parameters were considered statistically significant when p < 0.05.

## Results

Thirty-nine patients were included in this study. Demographic and clinical characteristics of the patient cohort, along with patients who experienced GR2 + pneumonitis versus those who did not, are shown in Table 1. Patients who received consolidation ICI-therapy (n = 21) had similar characteristics to those who did not (Supplementary Table A1). In accordance with the guidelines, ICI-therapy was initiated within 42 days after RT in 14/21 (67%) patients with a median delay of 22 days. Delays in initiation of ICI were primarily related to clinical recovery and acute toxicity from radiation treatment. Patients had median follow-up of 16.6 months (7–50.6 months), with 29/39 exceeding one-year of follow-up.

Table 2 shows that on univariate analysis of the clinical characteristics, and anatomic/functional lung dosimetry parameters, only the presence of baseline chronic obstructive pulmonary disease (COPD) appeared as a significant predictor for radiation pneumonitis, (HR 4.59 [95% CI: 1.69–12.49], p = 0.003). With a larger sample size, the hazard ratio estimate may be more precise with correspondingly narrower confidence interval. Multivariate LASSO-Cox proportional hazards model also showed that COPD independently associated with the development of pneumonitis when including clinical features only (HR 2.33) and when including clinical and anatomical/functional dosimetric features (HR 2.18). The predictive performance of the COPD-only model (c-index 0.69, 95% CI: 0.59–0.80) was used as a benchmark for multivariable prediction models that incorporated pre-treatment lung perfusion radiomic features.

**Table 2:** Univariable and multivariable associations of clinical factors and lung dosimetric parameters with pneumonitis risk in the setting of functional lung avoidance radiation treatment planning

Clinical feature	HR	Univariable		LASSO HR^a^		
		(95% CI)	P-value	Clinical only	Dosimetry only	Clinical + Dosimetry
Male	1.31	(0.49- 3.50)	0.59	–		–
Age, per 10-year increase	1.67	(0.84- 3.29)	0.14	–		–
COPD	4.59	(1.69- 12.49)	0.003	2.33		2.18
Stage IIIb	0.55	(0.18–1.72)	0.31	–		–
PBT	1.05	(0.39–2.81)	0.92	–		–
Chemotherapy Carboplatin + paclitaxel	1.69	(0.59–4.88)	0.33	–		–
ICI	1.71	(0.58–5.01)	0.33	–		–
Dosimetric Feature						
MLD, per 1-SD increase	1.24	(0.75–2.06)	0.41		–	–
V20, per 1-SD increase	1.29	(0.78–2.13)	0.33		1.02	–
sqrt(pMLD), per 1-SD increase	1.07	(0.67–1.73)	0.77		–	–
sqrt(pV20), per 1-SD increase	1.06	(0.66–1.71)	0.81		–	–
pF20, per 1-SD increase	1.13	(0.71–1.80)	0.61		–	–

*HR* hazard ratio, *LASSO* least absolute shrinkage and selection operator, *COPD* chronic pulmonary obstructive disease, *PBT* proton-beam therapy; *ICI* immune checkpoint inhibitor, *MLD* mean lung dose, *V20* volume of lung receiving > 20 Gy, *pMLD* perfused mean lung dose, *pV20* perfused lung volume receiving ≥ 20 Gy, *pF20* fraction of integral lung function receiving ≥ 20 Gy

^a^HR from LASSO-Cox model; a dash (-) indicates the corresponding feature was included in the model but the LASSO did not select it in the final model (HR = 1); blank cells indicate the features were not included in the LASSO-Cox model

**Fig. 2:**
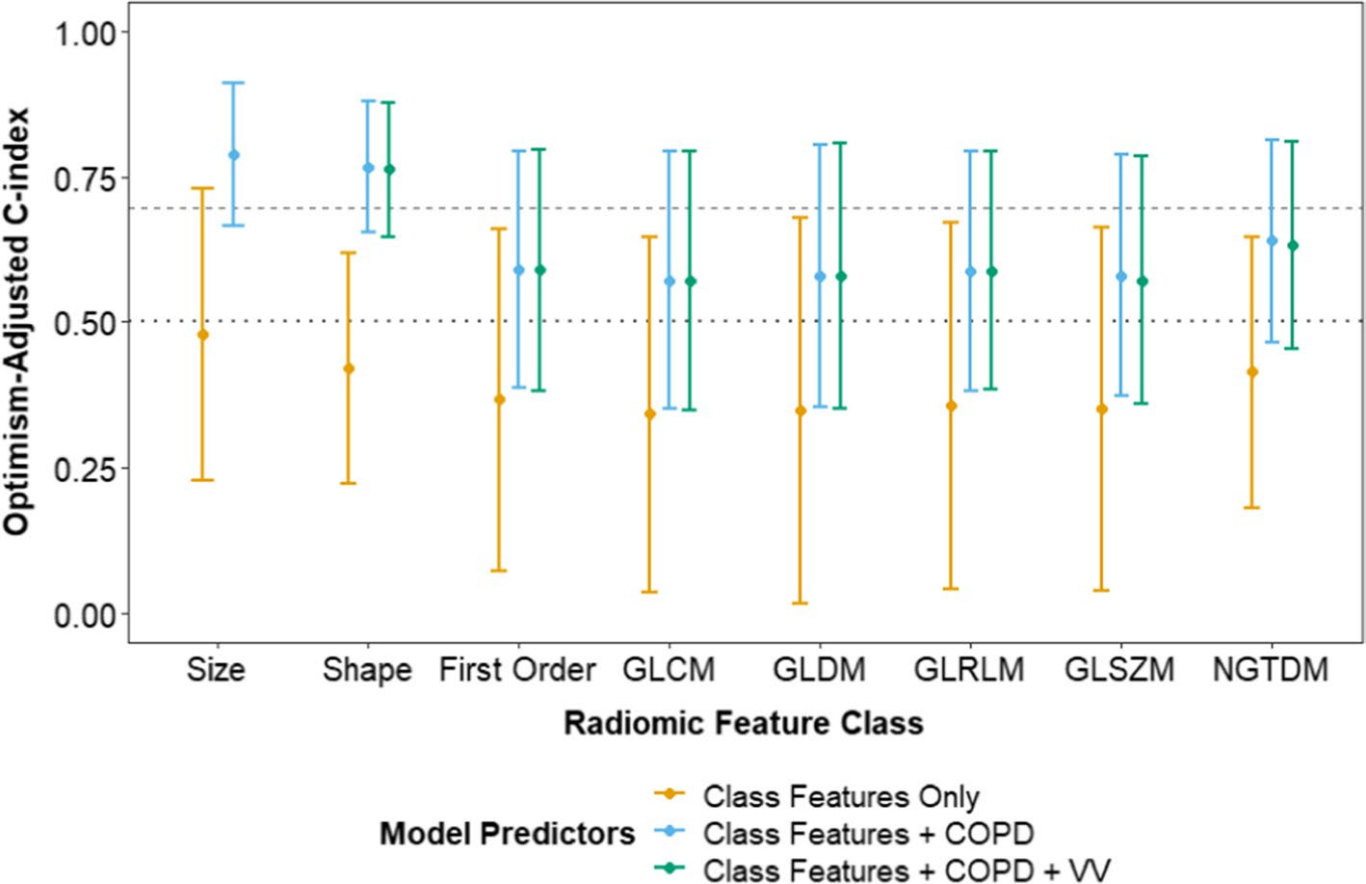
Performance of LASSO-Cox multivariable models for pneumonitis prediction based on lung radiomic features only (orange) or in conjunction with chronic obstructive pulmonary disease (COPD) (blue) or COPD and voxel volume (VV) (green) as added features. Each class of radiomic features was considered separately. The horizontal dotted line indicates the null value (c-index = 0.5) and the horizontal dashed line indicates the benchmark COPD-only model (c-index = 0.69). GLCM = gray-level co-occurance matrix; GLDM = gray-level dependence matrix; GLRLM = gray-level run-length matrix; GLSZM = gray-level size zone matrix; NGTDM = neighboring gray-tone difference matrix

Fig. 2 shows the LASSO-multivariable models of time-varying pneumonitis risk from pre-treatment lung perfusion radiomics features independently or with other factors such as COPD and lung volume. Models including only the radiomics features (orange points) with each feature class could not discriminate between patients with and without pneumonitis (c-index 0.34–0.48). However, when perfused lung radiomics shape (c-index 0.77 [95% CI: 0.65–0.88], p = 0.27 vs. benchmark COPD-only model) and size feature classes (c-index 0.79 [95% CI: 0.66–0.91], p = 0.26 vs. benchmark COPD-only model) were combined with COPD (blue points) the discrimination of time-varying pneumonitis risk improved but did not reach statistical significance compared to benchmark models. The addition of lung voxel volume to perfusion lung radiomics features and COPD (green points) had little impact on discrimination of time-varying pneumonitis risk (Fig. 2).

**Fig. 3:**
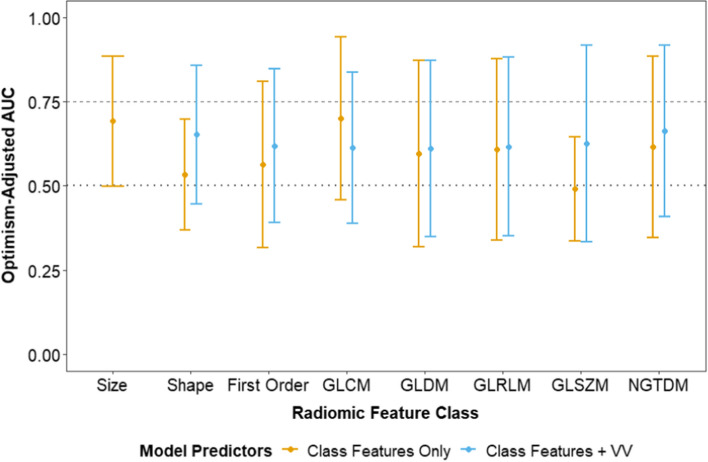
Performance of LASSO-logistic multivariable models for chronic obstructive pulmonary disease (COPD) prediction based on lung radiomic features only (orange) or in conjunction with voxel volume (VV) (blue) as an added feature. GLCM = gray-level co-occurance matrix; GLDM = gray-level dependence matrix; GLRLM = gray-level run-length matrix; GLSZM = gray-level size zone matrix; NGTDM = neighboring gray-tone difference matrix

Since COPD was the dominant risk factor for pneumonitis, we investigated its correlation to radiomic features. Figure 3 shows the LASSO logistic regression models for COPD prediction based on perfused lung radiomics features. The models included radiomic feature classes separately. COPD was marginally significantly associated with perfused lung radiomics size features (AUC 0.69 [95% CI: 0.50–0.89], p = 0.051) but not with the features in the other radiomic feature classes (AUC 0.36–0.70, p > 0.11 for all). Since COPD largely correlated to radiomic size feature class, we also examined a model with lung voxel volume as the only predictor (AUC 0.75 [95% CI: 0.59–0.91]) and added lung voxel volume to each class-specific model (blue points in Fig. 3). While lung voxel volume alone was significantly associated with COPD (p = 0.002), none of the models which combined voxel volume with perfused lung radiomic features were statistically significantly predictive of COPD (AUC 0.61–0.66, p > 0.15 for all). As shown in Table 3, perfused lung radiomic texture features were highly correlated with lung volume (adj R^2^ = 0.84–1.00).

**Table 3:** Correlation of lung voxel volume with perfused lung radiomic features within each feature class

Feature class	Number of features in class	Correlation between volume and all features within a class	
		adj.R^2^	P-value
Size^a^	9	1.00	< 0.001
Shape	4	0.93	< 0.001
First Order	17	1.00	< 0.001
GLCM	23	0.87	< 0.001
GLDM	14	0.99	< 0.001
GLRLM	16	1.00	< 0.001
GLSZM	16	0.98	< 0.001
NGTDM	5	0.84	< 0.001

^a^Voxel volume is excluded from the set of size features in the calculations

## Discussion

To our knowledge, we present the first study exploring the role of MAA-SPECT lung perfusion radiomics for pneumonitis risk stratification. A unique cohort of LA-NSCLC patients enrolled on the FLARE-RT protocol (NCT02773238) and received chemoradiation guided by functional lung avoidance planning and selective dose escalation based on mid-treatment fluorodeoxyglucose-PET response status, with approximately half of patients receiving consolidation anti-PD-L1 ICI-therapy. Identifying novel biomarkers beyond conventional clinical factors and radiation dosimetry is especially relevant in the modern cancer immunotherapy era. In this cohort, patients received ICI starting a few weeks after radiation treatment completion for up to a year thereafter, creating overlapping onset periods of radiation-induced and ICI-induced pneumonitis. We evaluated clinical and lung dosimetric parameters independently, as well as in combination with perfused lung radiomic features derived from MAA-SPECT for overall pneumonitis risk stratification. Discrimination between radiation and ICI-induced pneumonitis may be further addressed by ongoing clinical trials testing combinations of radiation and ICI administration, including neo-adjuvant, concurrent, adjuvant regimens.

Moran et al. evaluated GLCM-texture and first-order features to assess their ability to distinguish between oncologist-defined post-SBRT lung injury, with GLCM features outperforming first-order features [44]. Cunliffe et al. observed CT-texture changes in high dose regions that correlated strongly to radiation pneumonitis status, while dosimetric parameters failed to show any association. Krafft et al. reported that the addition of CT-based texture features along with clinical and dosimetric features improved the performance of pneumonitis prediction models [43]. Our investigation revealed that none of the radiomics features classes showed promise in predicting risk of pneumonitis independently. However, when radiomics shape and size class features were combined with COPD, the discrimination of time-varying risk prediction of pneumonitis improved (c-index = 0.77–0.79) although it did not reach statistical significance. Similar to cancer radiomics where tumour volume is a confounding feature [58], many perfused lung radiomic features showed marked dependencies with volume of the lung, which is the organ at risk in our study, suggesting the radiomic features may serve as size surrogates rather than independent predictors of pneumonitis risk.

In the era of ICI consolidation therapy for LA-NSCLC, few studies have evaluated clinical and dosimetric risk factors for pneumonitis. Shaverdian et al. found that validated pulmonary toxicity models underestimated the incidence of pneumonitis in the setting of consolidation durvalumab ICI [47]. Although current smoking is discussed as a possible protective factor [22, 59], we could not include smoking status in the regression analyses shown in Table 2 due to the small number of current smokers (n = 4) and never smokers (n = 5). PD-L1 is another known risk factor for pneumonitis in patients treated with ICI [4, 16]. However, PD-L1 expression was only available in 24/39 (62%) subjects and in 10/21 (48%) who underwent ICI therapy. Each group (< 1%, 1–49%, > 50%) had only 4–6 patients (Table1). Therefore, we were not able to include PD-L1 expression in the univariable and multivariable analyses in Table 2.

Inoue et al. reported that clinical and dosimetric parameters were not significant predictors of pneumonitis in LA-NSCLC treated with the chemoradiation and durvalumab regimen [48]. Our findings in the FLARE-RT cohort were concordant with these studies, as our anatomic and perfused lung dosimetry failed to predict pneumonitis incidence. We observed that COPD or pre-existing emphysema was the dominant risk factor for the development of pneumonitis in this cohort of patients. These results are similar to the study reported by Zhou et al. who observed that pulmonary emphysema was a risk factor for NSCLC patients with squamous cell carcinoma [12]. The main difference in our study is the inclusion of ICI therapy in the consolidative and adjuvant setting following RT, which is a modulator of overall pneumonitis risk. Patients with COPD have been known to exhibit increases in lung volume due to limited expiratory airflow [60, 61]. In this study, we found that COPD was more prevalent in patients with larger lung volumes, which is consistent with the lung physiology as reported in the studies above.

Our study has some limitations. In this preliminary discovery phase, we report on a modest patient cohort, with only half of patients receiving ICI-therapy. Nonetheless, very few reports are available on the incidence of immunotherapy-related pneumonitis following chemoradiation for locally advanced non-small cell lung cancer, and none incorporated functional lung avoidance planning techniques or functional lung radiomics. With 16 pneumonitis events we expected to be able to reliably include two to three predictors in non-penalized models [62]. To reliably accommodate more predictors simultaneously, we utilized the LASSO, a penalized regression technique which can stabilize the models [63, 64] However, the number of events does ultimately affect statistical power, so it is possible some risk factors with relatively weak associations were not detected.

Also, our study lacks external validation of the functional lung radiomic pneumonitis prediction model. However, as we continue treating our patients with chemoradiation and ICI-therapy, there is opportunity to validate the findings of this study in a larger cohort of patients to further elucidate the influence of COPD, perfused lung dosimetry, and perfused lung radiomics on pneumonitis risk. Once validated, the future models may inform the personalised initiation of immunotherapy in patients based in part on risk of pneumonitis, with for example concurrent radiation and ICI in low-risk patients, or the use of prophylactic corticosteroids for high-risk patients. These concepts can be explored in future clinical trials.

Lastly, while we relied on SPECT CNTS data, the latest generation SPECT scanners will enable calculation of SPECT standardized uptake values (SUV) that are analogous to PET SUV, thus facilitating quantitative SPECT radiomics. Future quantitative SPECT radiomic approaches should consider the effects of total lung volume and perfused lung available for MAA tracer distribution on SPECT SUV calculations.

## Conclusion

As immune modulating therapies grow more influential in the management of unresectable locally advanced non-small cell lung cancer, modelling of pneumonitis risk secondary to radiation and systemic therapy requires investigation of biomarkers and factors beyond conventional radiation dosimetry. Combinations of clinical, dosimetric, and radiomic features may support personalized treatments to mitigate the risk of developing pneumonitis. In this work, we identified the presence of underlying pulmonary disease as a strong predictor of radiation and immune-mediated pneumonitis risk and observed that perfused lung radiomics may represent lung volume surrogates for non-invasive monitoring of pulmonary disease rather than independent predictors of pneumonitis risk. Validation of these models can potentially personalise the initiation of immunotherapy in the adjuvant, neoadjuvant, or concurrent settings based on risk of pneumonitis. Further study of clinical, dosimetric, and radiomic feature combinations for radiation and immune-mediated pneumonitis risk stratification in a larger patient population is warranted.

## Supplementary Information

Additional file1 (DOCX 36 KB)

## Data Availability

The datasets from the current study are not publicly available prior to reporting of mature outcomes from the parent clinical trial. Requests for data and materials should be sent to the corresponding author.
