# Supplementary material for: Radiation and immune checkpoint inhibitor-mediated pneumonitis risk stratification in patients with locally advanced non-small cell lung cancer: role of functional lung radiomics?
Source: Discov Oncol. 2022 Sep 1;13:85. doi: 10.1007/s12672-022-00548-4 (PMC9437196; doi:10.1007/s12672-022-00548-4)
Supplement: Supplementary file 1 — Additional file1 (DOCX 36 KB) [file 12672_2022_548_MOESM1_ESM.docx]

**Appendix**

**Table A1:**Patient demographics and clinical characteristics stratified by immune checkpoint inhibitor therapy status.

|  | **ICI-Therapy** | |  |
| --- | --- | --- | --- |
| **Characteristics** | **No**  **(N = 18)** | **Yes**  **(N = 21)** | **P-value*** |
| Age, years | 63 (51 - 75) | 64 (48 - 78) | 0.66 |
| Gender |  |  | 0.52 |
| Male | 7 (39) | 11 (52) |  |
| Female | 11 (61) | 10 (48) |  |
| Clinical stage |  |  | 0.95 |
| IIB | 1 (6) | 0 (0) |  |
| IIIA | 8 (44) | 11 (52) |  |
| IIIB | 6 (33) | 7 (33) |  |
| Recurrence | 3 (17) | 3 (14) |  |
| Treatment modality |  |  | 0.20 |
| IMRT/VMAT | 11 (61) | 8 (38) |  |
| PBT | 7 (39) | 13 (62) |  |
| NSCLC histology |  |  | 0.50 |
| Squamous cell carcinoma | 6 (33) | 8 (38) |  |
| Adenocarcinoma | 12 (67) | 11 (52) |  |
| NOS | 0 (0) | 2 (10) |  |
| COPD |  |  | >0.99 |
| Yes | 6 (33) | 7 (33) |  |
| No | 12 (67) | 14 (67) |  |
| Chemotherapy |  |  | 0.19 |
| Carboplatin-paclitaxel | 13 (72) | 10 (48) |  |
| Others | 5 (28) | 11 (52) |  |
| Smoking status |  |  | 0.21 |
| Non-smoker | 4 (22) | 1 (5) |  |
| Former | 13 (72) | 17 (81) |  |
| Current | 1 (6) | 3 (14) |  |
| Mid-treatment PET response |  |  |  |
| Responder | 10 (56) | 15 (71) | 0.34 |
| Non-responder | 8 (44) | 6 (29) |  |

Values are median (range) or no. (%);

IMRT = intensity-modulated radiation therapy; VMAT = volumetric modulated arc therapy; PBT = proton-beam therapy; NSCLC = non-small cell lung cancer NOS = not otherwise specified; COPD = chronic obstructive pulmonary disease; ICI = immune-checkpoint inhibitor;

*Mann-Whitney test or Fisher’s exact test comparing the patients who did or did not undergo ICI-therapy.

**Table A2:** LASSO-Cox models for pneumonitis.

|  |  |  | **Models (Hazard Ratios)** | | |
| --- | --- | --- | --- | --- | --- |
|  | **Feature** | **Transformation** | **Class Features**  **Only** | **Class Features +**  **COPD** | **Class Features +**  **COPD + VV** |
| **Size Class Models** | COPD |  |  | 39.21 | 39.21 |
|  | VoxelVolume | log | - | 0.22 | 0.22 |
|  | Maximum3DDiameter | log | - | 1.87 | 1.87 |
|  | MeshVolume | log | - | - | - |
|  | MajorAxisLength | log | - | - | - |
|  | LeastAxisLength | log | - | 2.43 | 2.43 |
|  | Maximum2DDiameterSlice | log | - | 0.18 | 0.18 |
|  | SurfaceArea | log | 1.20 | 6.49 | 6.49 |
|  | MinorAxisLength | log | - | 14.49 | 14.49 |
|  | Maximum2DDiameterColumn | log | - | - | - |
|  | Maximum2DDiameterRow | log | - | 0.03 | 0.03 |
| **Shape Class Models** | COPD |  |  | 8.65 | 8.65 |
|  | VoxelVolume | log |  |  | - |
|  | Sphericity |  | - | 0.88 | 0.88 |
|  | Elongation |  | - | 1.60 | 1.60 |
|  | SurfaceVolumeRatio |  | - | 2.13 | 2.13 |
|  | Flatness |  | - | - | - |
| **First Order Feature Models** | COPD |  |  | 2.87 | 2.87 |
|  | VoxelVolume | log |  |  | - |
|  | InterquartileRange |  | - | - | - |
|  | Skewness |  | - | - | - |
|  | Uniformity |  | - | - | - |
|  | Median | log | - | - | - |
|  | Energy | log | - | - | - |
|  | RobustMeanAbsoluteDeviation | log | - | - | - |
|  | MeanAbsoluteDeviation | log | - | - | - |
|  | Maximum | log | - | - | - |
|  | RootMeanSquared | log | - | - | - |
|  | 90Percentile | log | - | - | - |
|  | Minimum |  | - | - | - |
|  | Entropy | log | - | - | - |
|  | Range | log | - | - | - |
|  | Variance | log | - | - | - |
|  | 10Percentile | log | - | - | - |
|  | Kurtosis |  | - | - | - |
|  | Mean | log | - | - | - |
| **GLCM Models** | COPD |  |  | 2.87 | 2.87 |
|  | VoxelVolume | log |  |  | - |
|  | SumAverage | log | - | - | - |
|  | JointEntropy |  | - | - | - |
|  | ClusterShade | cbrt | - | - | - |
|  | MaximumProbability | log | - | - | - |
|  | Idmn |  | - | - | - |
|  | JointEnergy | log | - | - | - |
|  | Contrast | log | - | - | - |
|  | DifferenceEntropy |  | - | - | - |
|  | InverseVariance |  | - | - | - |
|  | DifferenceVariance | log | - | - | - |
|  | Idn |  | - | - | - |
|  | Idm |  | - | - | - |
|  | Correlation |  | - | - | - |
|  | Autocorrelation | log | - | - | - |
|  | SumEntropy |  | - | - | - |
|  | MCC |  | - | - | - |
|  | SumSquares | log | - | - | - |
|  | ClusterProminence | log | - | - | - |
|  | Imc2 |  | - | - | - |
|  | Imc1 |  | - | - | - |
|  | DifferenceAverage |  | - | - | - |
|  | Id |  | - | - | - |
|  | ClusterTendency | log | - | - | - |
| **GLDM Models** | COPD |  |  | 2.33 | 2.33 |
|  | VoxelVolume | log |  |  | - |
|  | GrayLevelVariance | log | - | - | - |
|  | HighGrayLevelEmphasis | log | - | - | - |
|  | DependenceEntropy |  | - | - | - |
|  | DependenceNonUniformity |  | - | - | - |
|  | GrayLevelNonUniformity1 |  | - | - | - |
|  | SmallDependenceEmphasis |  | - | - | - |
|  | SmallDependenceHighGrayLevelEmphasis | log | - | - | - |
|  | DependenceNonUniformityNormalized |  | - | - | - |
|  | LargeDependenceEmphasis | log | - | - | - |
|  | LargeDependenceLowGrayLevelEmphasis | log | - | - | - |
|  | DependenceVariance | log | - | - | - |
|  | LargeDependenceHighGrayLevelEmphasis |  | - | - | - |
|  | SmallDependenceLowGrayLevelEmphasis |  | - | - | - |
|  | LowGrayLevelEmphasis | log | - | - | - |
| **GLRLM Models** | COPD |  |  | 2.61 | 2.61 |
|  | VoxelVolume | log |  |  | - |
|  | ShortRunLowGrayLevelEmphasis | log | - | - | - |
|  | GrayLevelVariance | log | - | - | - |
|  | LowGrayLevelRunEmphasis | log | - | - | - |
|  | GrayLevelNonUniformityNormalized |  | - | - | - |
|  | RunVariance | log | - | - | - |
|  | GrayLevelNonUniformity2 |  | - | - | - |
|  | LongRunEmphasis | log | - | - | - |
|  | ShortRunHighGrayLevelEmphasis | log | - | - | - |
|  | RunLengthNonUniformity | log | - | - | - |
|  | ShortRunEmphasis |  | - | - | - |
|  | LongRunHighGrayLevelEmphasis | log | - | - | - |
|  | RunPercentage |  | - | - | - |
|  | LongRunLowGrayLevelEmphasis | log | - | - | - |
|  | RunEntropy | log | - | - | - |
|  | HighGrayLevelRunEmphasis | log | - | - | - |
|  | RunLengthNonUniformityNormalized |  | - | - | - |
| **GLSZM Models** | COPD |  |  | 2.47 | 2.47 |
|  | VoxelVolume | log |  |  | - |
|  | GrayLevelVariance | log | - | - | - |
|  | ZoneVariance | log | - | - | - |
|  | GrayLevelNonUniformityNormalized |  | - | - | - |
|  | SizeZoneNonUniformityNormalized |  | - | - | - |
|  | SizeZoneNonUniformity |  | - | - | - |
|  | GrayLevelNonUniformity3 |  | - | - | - |
|  | LargeAreaEmphasis | log | - | - | - |
|  | SmallAreaHighGrayLevelEmphasis | log | - | - | - |
|  | ZonePercentage |  | - | - | - |
|  | LargeAreaLowGrayLevelEmphasis | log | - | - | - |
|  | LargeAreaHighGrayLevelEmphasis | log | - | - | - |
|  | HighGrayLevelZoneEmphasis | log | - | - | - |
|  | SmallAreaEmphasis |  | - | - | - |
|  | LowGrayLevelZoneEmphasis |  | - | - | - |
|  | ZoneEntropy |  | - | - | - |
|  | SmallAreaLowGrayLevelEmphasis |  | - | - | - |
| **NGTDM Models** | COPD |  |  | 2.74 | 2.74 |
|  | VoxelVolume | log |  |  | - |
|  | Coarseness |  | - | - | - |
|  | Complexity | log | - | - | - |
|  | Strength | log | - | - | - |
|  | Contrast | log | - | - | - |
|  | Busyness |  | - | - | - |

Values are hazard ratios unless otherwise specified; a dash indicates the feature was included in the model but the LASSO did not select it; a blank cell indicates the feature was not included at all in the model;

COPD = chronic obstructive pulmonary disease; VV = Voxel Volume; GLCM = gray-level co-occurance matrix; GLDM = gray-level dependence matrix; GLRLM = gray-level run-length matrix; GLSZM = gray-level size zone matrix; NGTDM = neighboring gray-tone difference matrix.

**Table A3** : LASSO-logistic models for COPD.

|  |  |  | **Models (Odds Ratios)** | |
| --- | --- | --- | --- | --- |
|  | **Feature** | **Transformation** | **Class Features**  **Only** | **Class Features +**  **COPD** |
| **Size Class Models** | VoxelVolume | log | - | - |
|  | Maximum3DDiameter | log | - | - |
|  | MeshVolume | log | - | - |
|  | MajorAxisLength | log | - | - |
|  | LeastAxisLength | log | 2.04 | 2.04 |
|  | Maximum2DDiameterSlice | log | - | - |
|  | SurfaceArea | log | - | - |
|  | MinorAxisLength | log | - | - |
|  | Maximum2DDiameterColumn | log | 1.11 | 1.11 |
|  | Maximum2DDiameterRow | log | - | - |
| **Shape Class Models** | VoxelVolume | log |  | 1.55 |
|  | Sphericity |  | - | - |
|  | Elongation |  | - | - |
|  | SurfaceVolumeRatio |  | - | - |
|  | Flatness |  | - | - |
| **First Order Feature Models** | VoxelVolume | log |  | 1.57 |
|  | InterquartileRange |  | - | - |
|  | Skewness |  | - | - |
|  | Uniformity |  | - | - |
|  | Median | log | 0.84 | - |
|  | Energy | log | - | - |
|  | RobustMeanAbsoluteDeviation | log | - | - |
|  | MeanAbsoluteDeviation | log | - | - |
|  | Maximum | log | - | - |
|  | RootMeanSquared | log | - | - |
|  | 90Percentile | log | - | - |
|  | Minimum |  | - | - |
|  | Entropy | log | - | - |
|  | Range | log | - | - |
|  | Variance | log | - | - |
|  | 10Percentile | log | - | - |
|  | Kurtosis |  | 1.42 | 1.43 |
|  | Mean | log | - | - |
| **GLCM Models** | VoxelVolume | log |  | 1.49 |
|  | SumAverage | log | - | - |
|  | JointEntropy |  | - | - |
|  | ClusterShade | cbrt | - | - |
|  | MaximumProbability | log | - | - |
|  | Idmn |  | - | - |
|  | JointEnergy | log | 1.31 | - |
|  | Contrast | log | - | - |
|  | DifferenceEntropy |  | - | - |
|  | InverseVariance |  | - | - |
|  | DifferenceVariance | log | - | - |
|  | Idn |  | - | - |
|  | Idm |  | - | - |
|  | Correlation |  | - | - |
|  | Autocorrelation | log | - | - |
|  | SumEntropy |  | - | - |
|  | MCC |  | - | - |
|  | SumSquares | log | - | - |
|  | ClusterProminence | log | 9.58 | - |
|  | Imc2 |  | 0.21 | - |
|  | Imc1 |  | - | - |
|  | DifferenceAverage |  | 0.05 | - |
|  | Id |  | - | - |
|  | ClusterTendency | log | - | - |
| **GLDM Models** | VoxelVolume | log |  | 1.55 |
|  | GrayLevelVariance | log | - | - |
|  | HighGrayLevelEmphasis | log | - | - |
|  | DependenceEntropy |  | - | - |
|  | DependenceNonUniformity |  | - | - |
|  | GrayLevelNonUniformity1 |  | 1.24 | - |
|  | SmallDependenceEmphasis |  | - | - |
|  | SmallDependenceHighGrayLevelEmphasis | log | - | - |
|  | DependenceNonUniformityNormalized |  | - | - |
|  | LargeDependenceEmphasis | log | - | - |
|  | LargeDependenceLowGrayLevelEmphasis | log | - | - |
|  | DependenceVariance | log | - | - |
|  | LargeDependenceHighGrayLevelEmphasis |  | - | - |
|  | SmallDependenceLowGrayLevelEmphasis |  | - | - |
|  | LowGrayLevelEmphasis | log | - | - |
| **GLRLM Models** | VoxelVolume | log |  | 1.61 |
|  | ShortRunLowGrayLevelEmphasis | log | - | - |
|  | GrayLevelVariance | log | - | - |
|  | LowGrayLevelRunEmphasis | log | - | - |
|  | GrayLevelNonUniformityNormalized |  | - | - |
|  | RunVariance | log | - | - |
|  | GrayLevelNonUniformity2 |  | - | - |
|  | LongRunEmphasis | log | - | - |
|  | ShortRunHighGrayLevelEmphasis | log | - | - |
|  | RunLengthNonUniformity | log | 1.33 | - |
|  | ShortRunEmphasis |  | - | - |
|  | LongRunHighGrayLevelEmphasis | log | - | - |
|  | RunPercentage |  | - | - |
|  | LongRunLowGrayLevelEmphasis | log | - | - |
|  | RunEntropy | log | - | - |
|  | HighGrayLevelRunEmphasis | log | - | - |
|  | RunLengthNonUniformityNormalized |  | - | - |
| **GLSZM Models** | VoxelVolume | log |  | 1.61 |
|  | GrayLevelVariance | log | - | - |
|  | ZoneVariance | log | - | - |
|  | GrayLevelNonUniformityNormalized |  | - | - |
|  | SizeZoneNonUniformityNormalized |  | - | - |
|  | SizeZoneNonUniformity |  | - | - |
|  | GrayLevelNonUniformity3 |  | - | - |
|  | LargeAreaEmphasis | log | - | - |
|  | SmallAreaHighGrayLevelEmphasis | log | - | - |
|  | ZonePercentage |  | - | - |
|  | LargeAreaLowGrayLevelEmphasis | log | - | - |
|  | LargeAreaHighGrayLevelEmphasis | log | - | - |
|  | HighGrayLevelZoneEmphasis | log | - | - |
|  | SmallAreaEmphasis |  | - | - |
|  | LowGrayLevelZoneEmphasis |  | - | - |
|  | ZoneEntropy |  | - | - |
|  | SmallAreaLowGrayLevelEmphasis |  | - | - |
| **NGTDM Models** | VoxelVolume | log |  | 1.63 |
|  | Coarseness |  | 0.66 | 0.93 |
|  | Complexity | log | - | - |
|  | Strength | log | - | - |
|  | Contrast | log | - | - |
|  | Busyness |  | - | - |

Values are odds ratios unless otherwise specified; a dash indicates the feature was included in the model but the LASSO did not select it; a blank cell indicates the feature was not included at all in the model;

COPD = chronic obstructive pulmonary disease; VV = Voxel Volume; GLCM = gray-level co-occurance matrix; GLDM = gray-level dependence matrix; GLRLM = gray-level run-length matrix; GLSZM = gray-level size zone matrix; NGTDM = neighboring gray-tone difference matrix.
